# Insights into gastric mixed adenoneuroendocrine carcinoma: a novel comparative study of clinicopathological features and survival outcomes

**DOI:** 10.3389/fendo.2025.1650314

**Published:** 2025-09-30

**Authors:** Huayong Tan, Jing Huang, Gaochun Xiao, Junzhi Liu, Huimin Li, Maojun Di, Yuanjian Hui

**Affiliations:** ^1^ Department of General Surgery, Taihe Hospital, Hubei University of Medicine, Shiyan, Hubei, China; ^2^ Tianjin Medical University Cancer Institute and Hospital, National Clinical Research Center for Cancer, Tianjin, China

**Keywords:** gastric, mixed adenoneuroendocrine carcinomas, prognosis, risk factors, clinicopathological characteristics

## Abstract

Mixed adenoneuroendocrine carcinoma (MANEC) is a rare and histologically complex malignancy. Due to its low incidence, data on gastric MANEC (G-MANEC) are limited, and its clinicopathological characteristics and prognosis remain poorly defined. In this study, we performed a retrospective analysis of 168 G-MANEC patients and identified a median age of 64.7 years at diagnosis. Cases were classified into adenocarcinoma (AC)-predominant and neuroendocrine carcinoma (NEC)-predominant subtypes based on histological composition, with large-cell NEC accounting for 69.9% of NEC-predominant tumors. Patterns of lymph node metastasis (LNM) included involvement of either a single component (AC or NEC) or both components. Survival analysis revealed a median overall survival (OS) of 24 months, with 1-, 3-, and 5-year survival rates of 72.6%, 39.5%, and 29.7%, respectively. Univariate and multivariate analyses identified tumor size, LNM, and histological subtype as independent prognostic factors. Compared with a cohort of 328 patients with pure gastric adenocarcinoma, G-MANEC cases exhibited distinct clinicopathological features—particularly in terms of tumor size, Ki-67 index, and LNM. Collectively, these findings underscore that G-MANEC is associated with significantly poorer overall survival than that of conventional gastric adenocarcinoma.

## Introduction

According to the 2022 WHO Classification of Endocrine and Neuroendocrine Neoplasms (NENs), these tumors are broadly divided into neuroendocrine tumors (NETs), neuroendocrine carcinomas (NECs), and mixed neuroendocrine–non-neuroendocrine neoplasms (MiNENs), based on morphological features, mitotic activity, and Ki-67 proliferation index (1–3). MiNENs are defined as neoplasms comprising both neuroendocrine and non-neuroendocrine components, with each accounting for at least 30% of the tumor volume. A specific subtype described in the 2010 WHO classification, known as mixed adeno-neuroendocrine carcinoma (MANEC), consists of poorly differentiated NEC and adenocarcinoma components meeting the same threshold (4). This study focuses on tumors containing both adenocarcinoma and NEC components with relatively low-grade features. For consistency and in line with most of the literature (4, 5), we use the term MANEC as defined in the 2010 WHO classification.

According to data from the Surveillance, Epidemiology, and End Results (SEER) program, Gastrointestinal Neuroendocrine Neoplasms (GI-NEN) are rare malignancies, with an annual incidence of 3.56 per 100,000 individuals in the United States, and similar rates observed between males and females (6, 7). Notably, their incidence has increased significantly over the past few decades, largely due to advances in diagnostic techniques (8). Despite these findings, the literature on GI-MANEC remains limited, primarily consisting of isolated case reports (9–14). This scarcity impedes the development of a comprehensive understanding of this malignancy, resulting in a significant lack of consensus regarding its clinicopathological characteristics, diagnostic criteria, and prognostic implications, particularly with respect to MANEC. The uncertainty surrounding MANEC raises critical questions about its nature in comparison to its pure counterparts- NEC and adenocarcinoma (AC) (15). It is still under investigation whether MANEC demonstrates more aggressive clinical behavior than these established tumor types, a factor that could significantly influence treatment decisions and patient outcomes (16, 17). Furthermore, the National Comprehensive Cancer Network (NCCN) has yet to develop specific treatment guidelines tailored to MANEC (18), reflecting a broader challenge in standardizing care for this rare malignancy. This gap in clinical guidelines highlights the urgent need for further research to clarify optimal management strategies. Additionally, the complex relationship between tumor grade, histological features, and patient prognosis remains ambiguous, complicating clinicians’ ability to predict outcomes effectively. A concerted effort is required to establish standardized diagnostic criteria, investigate the biological behavior of this malignancy, and develop targeted treatment protocols to enhance patient care and outcomes.

In this study, we comprehensively evaluated the clinicopathological data and clinical outcomes of patients diagnosed with G-MANEC by systematically analyzing tumor differentiation, staging, and patient demographics. We aimed to identify specific patterns influencing patient outcomes. Our findings may ultimately contribute to the development of more effective treatment protocols, enhancing overall survival rates for patients with this complex malignancy.

## Materials and methods

### Case selection

We retrospectively analyzed the clinical data of patients diagnosed with G-MANEC at our center, Wuhan Union Hospital and Tianjin Medical University Cancer Institute and Hospital from January 2008 to December 2022. The inclusion criteria were as follows: (1) patients pathologically diagnosed with G-MANEC; (2) patients who underwent D2 lymph node dissection with a postoperative pathological diagnosis. The exclusion criteria were as follows: (1) patients who had received preoperative adjuvant chemotherapy or radiotherapy; (2) patients without follow-up data or those who died during the postoperative period; and (3) cases lacking detailed descriptions of both tumor components. All patients underwent radical gastrectomy, classified as total, distal, or proximal according to tumor location. Lymph node dissection was performed in accordance with the Japanese gastric cancer treatment guidelines.

### Diagnosis and classification of G-MANEC

The histologic, immunohistochemical, and clinical features of the cases were reassessed. All cases met the current diagnostic criteria for G-MANEC, as defined by the 2022 WHO classification. G-MANEC is characterized as a malignant tumor containing at least 30% glandular epithelial cells alongside neuroendocrine cells (19, 20). This multicenter study incorporated ethical approvals from all participating centers. Each site’s Institutional Review Board (IRB) or equivalent ethical review committee approved the study protocol, ensuring ethical standards were upheld across all locations. The study design and conduct were standardized across centers to maintain consistency and ethical integrity. Informed consent was waived at all sites due to the retrospective nature of the study and the use of de-identified patient data, as approved by the respective IRBs or ethical review committees.

### Survival analysis and statistical analysis

Statistical analysis was conducted to evaluate the clinicopathological characteristics and prognosis of patients, focusing on factors such as age, gender, tumor location, size, TNM stage, tissue subtypes, lymph node metastasis (LNM) and distant metastasis, chemotherapy, Ki-67 index, differentiation, tumor component, and surgical resection. Differences among adenocarcinoma (AC) and G-MANEC were specifically analyzed. All analyses were performed using SPSS version 23.0. Continuous and categorical variables were compared using Student’s t-test, Pearson’s **χ**², or Fisher’s exact test, respectively. Kaplan–Meier analysis was utilized to calculate 1,3,5-year survival rates, with the log-rank test applied to compare survival curves. Additionally, binary logistic regression was employed to assess independent risk factors for 3-year survival. A p-value of less than 0.05 is considered statistically significant.

## Results

### Clinicopathologic characteristics of G-MANEC

**Figure 1:**
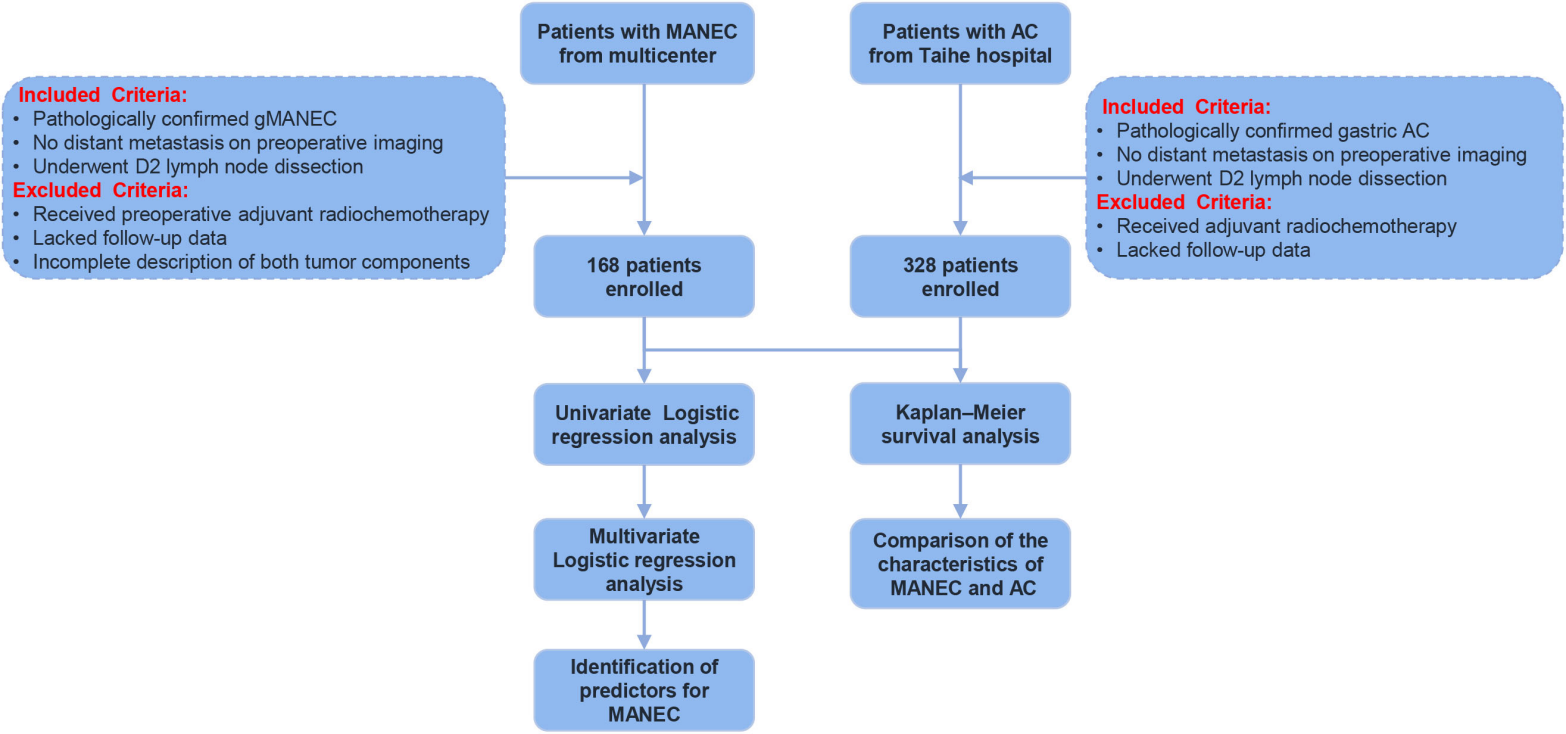
The flow chart of the study.

**Figure 1** presents the study flowchart. A total of 168 patients with G-MANEC were included in this cohort. The clinicopathological characteristics of the cohort are summarized in **Table 1**. The patients’ ages ranged from 34 to 81 years, with a mean age of 64.7 ± 8.0 years. Of these, 103 (61.3%) were male and 65 (38.7%) were female. Tumor locations varied, comprising 87 (51.8%) cases in the gastric cardia, 57 (33.9%) in the gastric body, and 24 (14.3%) in the gastric antrum. Curative resection (R0) was performed in 136 patients (81.0%), while 32 (19.0%) underwent incomplete resection (R1/R2). Tumor diameter exceeded 5cm in 100 patients (59.5%). Histological differentiation was categorized as poorly differentiated in 88 cases (52.5%) and well/moderately differentiated in 80 (47.5%). T-stage distribution included 22.6% T1/T2 and 77.4% T3/T4 tumors. Lymph node metastasis was present in 127 patients (75.6%), while 41 (24.4%) showed no nodal involvement. Tumor pTNM stages I/II and III/IV were observed in 58 (34.5%) and 100 (65.5%) patients, respectively. Using a Ki-67 index cut-off of 60%, 90 cases (53.6%) were positive and 78 (46.4%) were negative.

**Table 1:** Clinicopathological features of gastric MANEC.

Variable	Overall (n = 168)
Age (year)	64.7 ± 8.0
Gender	
Male	103 (61.3%)
BMI (kg/m^2^)	21.9 ± 3.4
Sizes (cm)	
<5	68 (40.5%)
≥5	100 (59.5%)
T stage	
T1+T2	58 (34.5%)
T3+T4	110 (65.5%)
Location	
Upper	87 (51.8%)
Middle	57 (33.9%)
Lower	24 (14.3%)
Ki-67 index	
<60%	78 (46.4%)
≥60%	90 (53.6%)
TNM stage	
I+II	69 (41.1%)
III+IV	99 (58.9%)
Surgical resection	
Complete	136 (81.0%)
Incomplete	32 (19.0%)
Lymph node metastasis	
No	72 (42.9%)
Yes	96 (57.1%)
AC metastasis	23 (34.3%)
NEC metastasis	24 (35.8%)
AC and NEC	20 (19.0%)
Distant metastases	
No	110 (65.5%)
Yes	58 (34.5%)
Vascular invasion	
No	67 (37.9%)
Yes	101 (60.1%)
Perineural invasion	
No	77 (45.8%)
Yes	91 (54.2%)
Tumor component	
**AC**	75 (44.6%)
DA	29 (38.7%)
MC	24 (32%)
PDA	22 (29.3%)
**NEC**	93 (55.4%)
Small cell	25 (30.1%)
Large cell	58 (69.9%)

Mean (SD); n (%); AC, adenocarcinoma; NEC, neuroendocrine carcinoma; DA, differentiated adenocarcinoma; MC, mucinous carcinoma; PDA, poorly differentiated adenocarcinoma.

**Figure 2:**
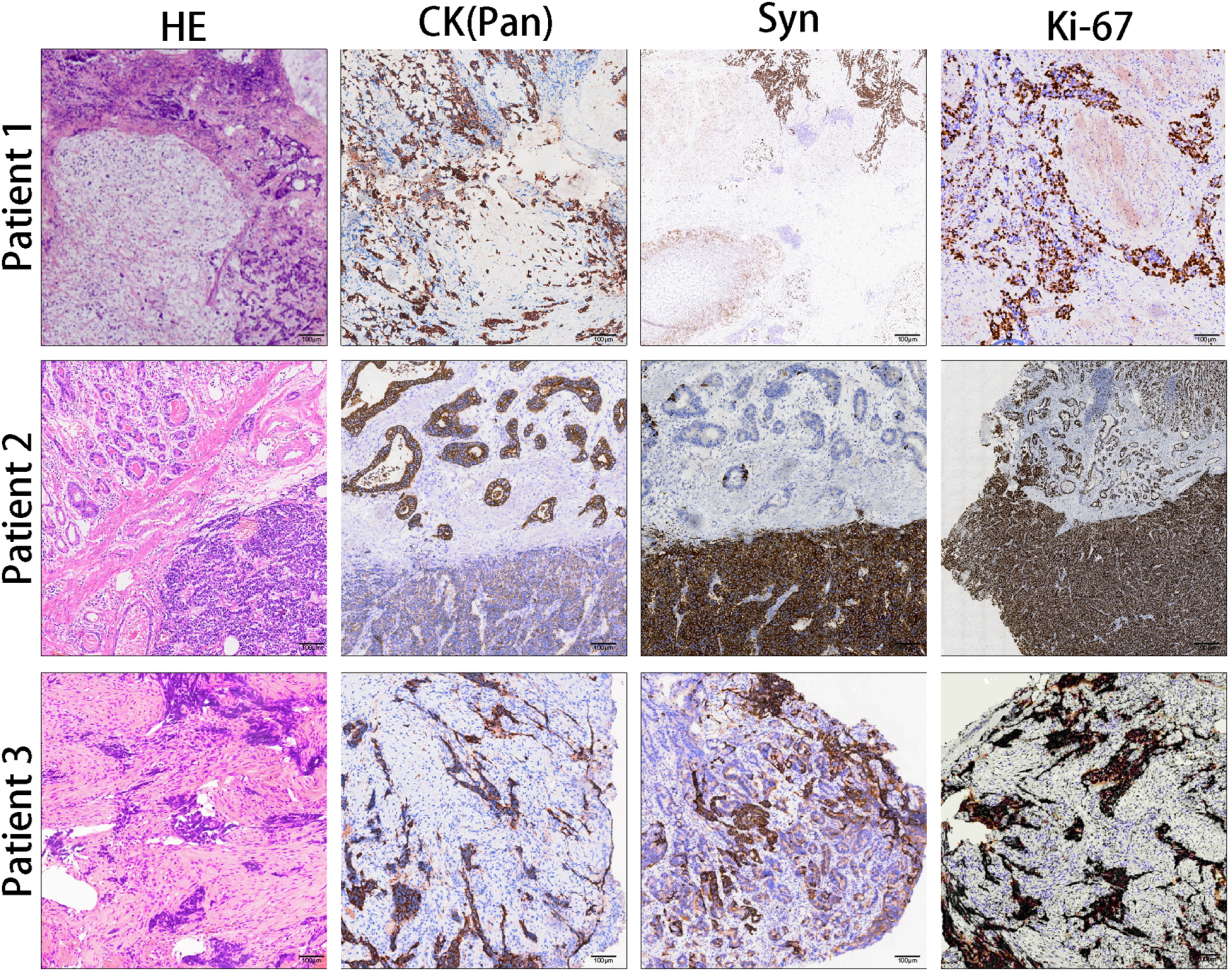
Comparative immunohistochemical analysis of three patient samples with different dominant histological types: Patient 1, adenocarcinoma (AC)-dominant; Patient 2, mixed type; Patient 3, neuroendocrine carcinoma (NEC)-dominant. The analyses include hematoxylin and eosin (HE) staining and immunohistochemical (IHC) staining for cytokeratin (CK, pan), synaptophysin (Syn), and Ki-67.

According to the different proportions and combination patterns of the AC and NEC components in tumors (greater than 50% of all tumors), G-MANEC can be divided into the NEC-dominant type and the AC-dominant type (**Figure 2**). We found 75 cases (44.6%) were diagnosed as adenocarcinoma-dominant type, and 93(55.4%) cases were NEC-dominant type. Among the NEC-dominant type tumors, most cases (58/83, 69.9%) were large cell NEC, and 25(25/83, 30.1%) cases were small cell NEC.

We further analyzed the status of lymph node metastasis and found that the lymph node metastasis patterns of G-MANEC are diverse, including the metastasis of adenocarcinoma or neuroendocrine carcinoma components, as well as mixed metastasis of both types. We further analyzed the status of lymph node metastasis and found that the patterns of metastasis in G-MANEC are diverse. Specifically, these patterns include metastases from adenocarcinoma components, neuroendocrine carcinoma components, and mixed components. Among the 96 samples analyzed, adenocarcinoma metastases accounted for 34.3%, while neuroendocrine carcinoma metastases represented 35.8%. Mixed component metastasis constituted 19% (**Table 1**).

### Prognosis of G-MANEC

**Figure 3:**
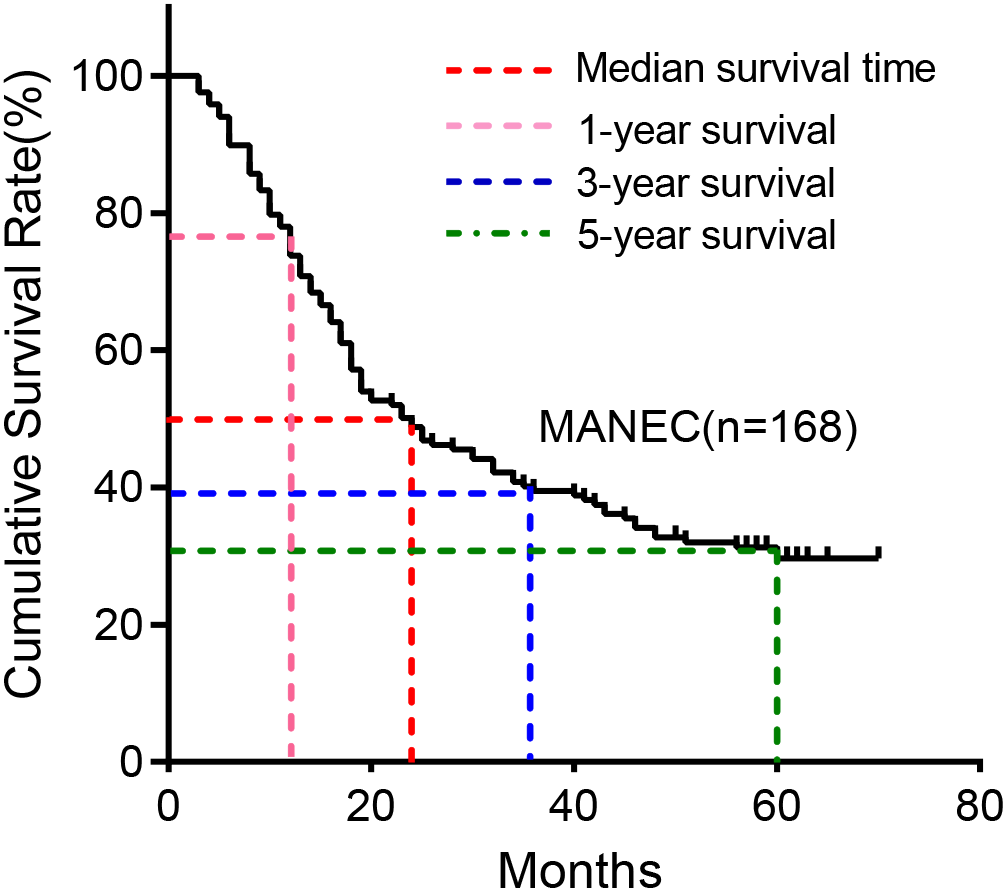
Kaplan-Meier survival plot of MANEC.

The follow-up data of 168 patients were analyzed for survival analysis. According to the analysis, the median follow-up period was 24 months ranging from 1 to 70 months. The 1,3,5-year survival rate was 72.62, 39.49 and 29.67, respectively (**Figure 3**).

**Figure 4:**
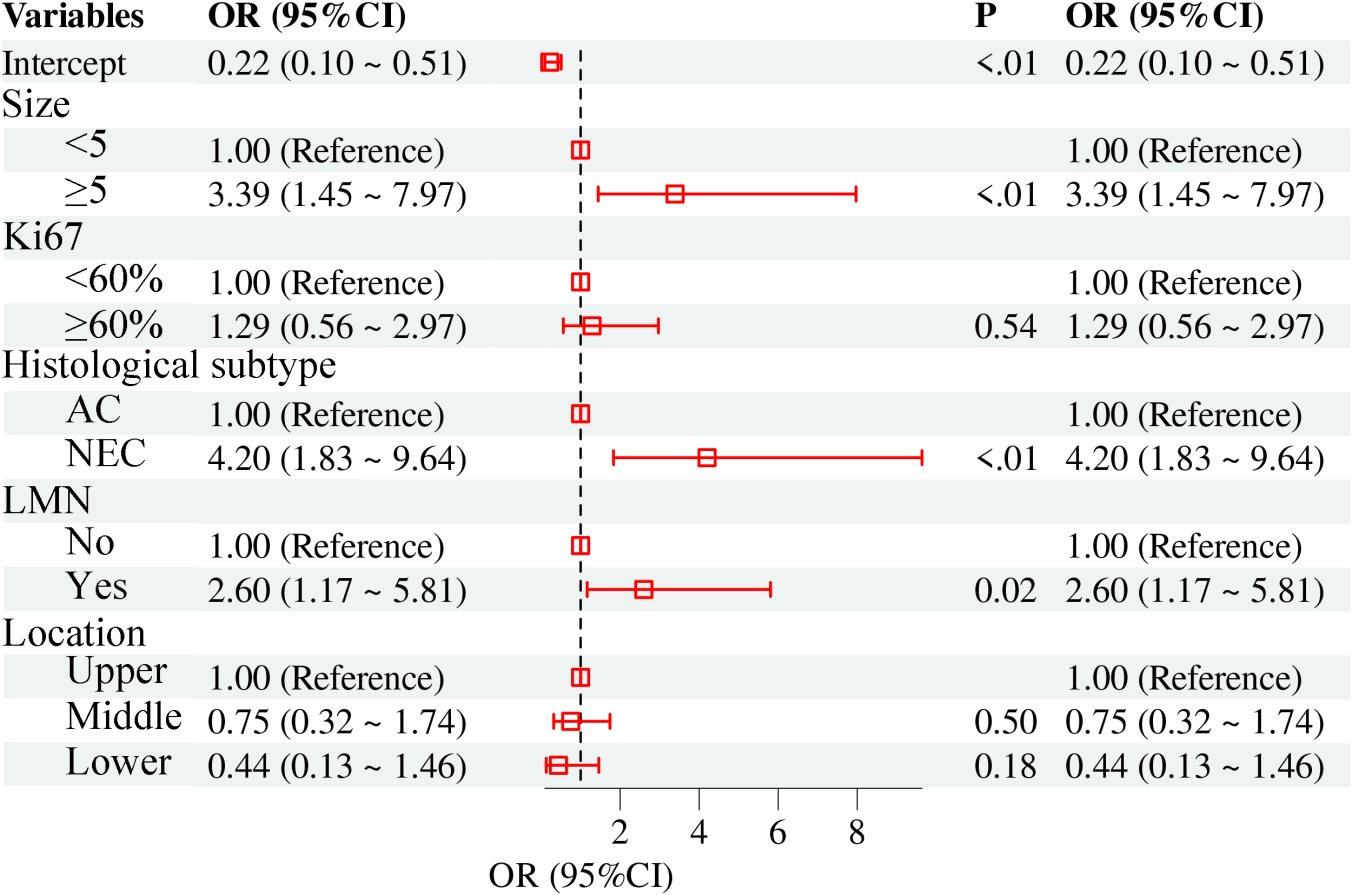
Forest plot showed the results of multivariate regression analysis.

We conducted univariate and multivariate analysis to identify prognostic predictors for patients, focusing on various clinical and demographic factors (**Table 2**). The results indicated that The univariate analysis identified tumor size ≥5 cm (OR=9.03, 95% CI: 4.44–18.36, P<0.01), Ki-67 index≥60% (OR=3.36, 95% CI: 1.77–6.40, P<0.01), NEC-dominant histology (OR=8.85, 95% CI: 4.37–17.96, P<0.01), LNM(OR=5.62, 95% CI: 2.87–10.99, P<0.01), and lower tumor location (OR=0.33, 95% CI: 0.13–0.85, P=0.02) as significant risk factors for G-MANEC. However, multivariate logistic regression analysis demonstrated that only tumor size ≥5 cm (OR=3.39, 95% CI: 1.45–7.97, P<0.01), NEC-dominant histology (OR=4.20, 95% CI: 1.83–9.64, P<0.01), and lymph node metastasis (OR=2.60, 95% CI: 1.17–5.81, P=0.02) remained independent predictors of G-MANEC after adjustment for confounding variables (**Figure 4**). Other factors, including Ki-67 index and tumor location, were not independently associated with G-MANEC in the multivariate model.

**Table 2:** Univariate and Multivariate analysis for the risk factors for MANEC.

Variables	Univariate analysis					Multivariate analysis				
	β	S.E	Z	*P*	OR (95%CI)	β	S.E	Z	*P*	OR (95%CI)
Age	-0.01	0.02	-0.47	0.64	0.99 (0.95 ~ 1.03)					
BMI	-0.03	0.05	-0.69	0.49	0.97 (0.88 ~ 1.06)					
Sizes										
<5					1.00 (Reference)					1.00 (Reference)
≥5	2.20	0.36	6.08	<.01	9.03 (4.44 ~ 18.36)	1.22	0.44	2.81	<.01	3.39 (1.45 ~ 7.97)
T stage, n (%)										
T1+T2					1.00 (Reference)					
T3+T4	0.39	0.32	1.22	0.22	1.47 (0.79 ~ 2.73)					
Ki-67										
<60%					1.00 (Reference)					1.00 (Reference)
≥60%	1.21	0.33	3.70	<.01	3.36 (1.77 ~ 6.40)	0.26	0.42	0.61	0.54	1.29 (0.56 ~ 2.97)
Surgical resection										
Incomplete					1.00 (Reference)					
Complete	0.52	0.42	1.25	0.21	1.69 (0.74 ~ 3.83)					
Histological subtype										
AC dominant					1.00 (Reference)					1.00 (Reference)
NEC dominant	2.18	0.36	6.04	<.01	8.85 (4.37 ~ 17.96)	1.44	0.42	3.39	<.01	4.20 (1.83 ~ 9.64)
LMN										
No					1.00 (Reference)					1.00 (Reference)
Yes	1.73	0.34	5.04	<.01	5.62 (2.87 ~ 10.99)	0.96	0.41	2.33	0.02	2.60 (1.17 ~ 5.81)
Gender										
Female					1.00 (Reference)					
Male	0.39	0.33	1.19	0.23	1.47 (0.78 ~ 2.79)					
Location										
Upper					1.00 (Reference)					1.00 (Reference)
Middle	-0.20	0.35	-0.57	0.57	0.82 (0.41 ~ 1.63)	-0.29	0.43	-0.68	0.50	0.75 (0.32 ~ 1.74)
Lower	-1.10	0.48	-2.31	0.02	0.33 (0.13 ~ 0.85)	-0.83	0.62	-1.35	0.18	0.44 (0.13 ~ 1.46)
Vascular invasion										
No					1.00 (Reference)					
Yes	0.15	0.32	0.47	0.64	1.16 (0.62 ~ 2.18)					
Perineural invasion										
No					1.00 (Reference)					
Yes	0.53	0.32	1.69	0.09	1.71 (0.92 ~ 3.17)					
Distant metastases										
No					1.00 (Reference)					
Yes	-0.02	0.33	-0.06	0.95	0.98 (0.51 ~ 1.87)					

OR, odds ratio; 95%CI, 95% confidence interval; BMI, body mass index; T stage, tumor stage; Ki-67, Ki-67; Index LMN, lymph node metastasis; AC, adenocarcinoma; NEC, neuroendocrine carcinoma.

Based on histological differentiation, the adenocarcinoma (AC) component is classified into differentiated adenocarcinoma (DA), mucinous carcinoma (MC), and poorly differentiated adenocarcinoma (PDA) (9). Our findings revealed that the 3-year survival rate of the DA group was higher than that of both the MC and PDA groups, with the PDA group showing significantly worse outcomes compared to the MC group. This indicates that the poorly differentiated glandular component may drive cancer progression in G-MANEC. Additionally, the neuroendocrine carcinoma (NEC) component is categorized into large cell and small cell types, with approximately 70% of NEC cases being large cell (58/93). No significant differences were observed in 3-year survival rates between the large and small cell groups (**Table 1**).

### Comparison of clinicopathological characteristics and prognosis between G-MANEC and gastric adenocarcinoma

**Figure 5:**
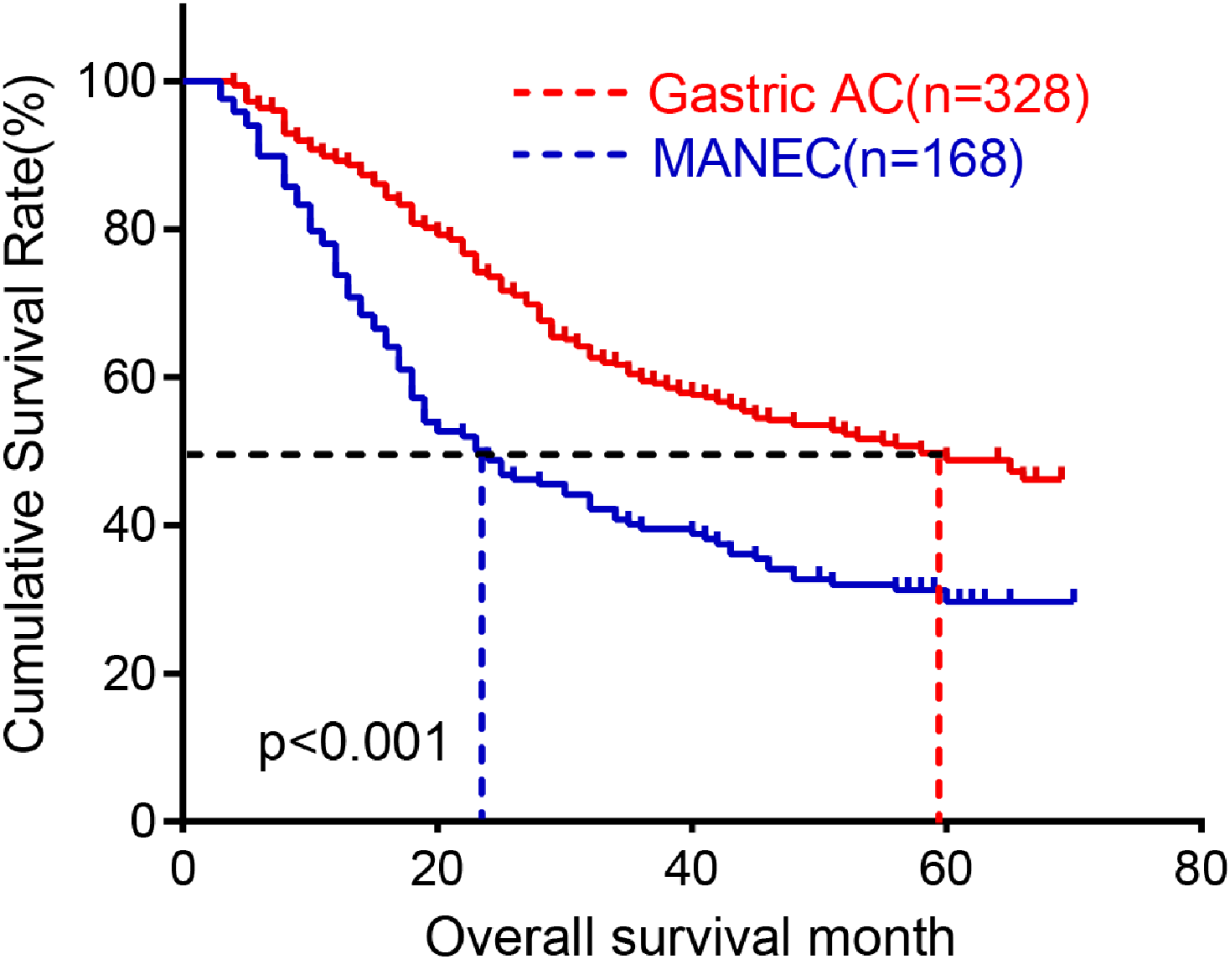
Comparison of the prognosis between gMANEC and gastric AC.

To minimize the potential confounding effects on prognostic comparisons, this study employed propensity score matching (PSM) to perform a 3:1 matched analysis between patients with G-MANEC and those with AC. Prior to matching, there were significant differences between the two groups in several baseline clinicopathological characteristics, including tumor size, T stage, Ki-67 index, LNM, sex, tumor location, and vascular invasion (all P < 0.05), indicating substantial selection bias. After PSM, 251 patients with AC and 129 with MANEC were included. Post-matching, differences between the two groups were no longer statistically significant for most variables, except for tumor size (P=0.045), Ki-67(P=0.025) and LNM (P=0.002). Moreover, all covariates exhibited standardized mean differences (SMD) below 0.2, suggesting good balance in baseline characteristics (**Table 3**). These findings indicate that tumor size, Ki-67 and LNM may be key factors contributing to the poor prognosis observed in patients with G-MANEC. To evaluate prognosis, we conducted a survival analysis comparing G-MANEC and AC patients. The results revealed that the overall survival (OS) rates at 1, 3, and 5 years for AC patients were 89.22%, 59.49%, and 48.85%, respectively, which were significantly better than those for G-MANEC patients. Specifically, G-MANEC patients exhibited a median overall survival of 24 months, compared to 56 months for AC patients (P < 0.001). These findings underscore that patients with G-MANEC have a poorer prognosis compared to those with AC (**Figure 5**).

**Table 3:** Comparison of clinicopathological characteristics between MANEC and AC.

Variable	Before PSM					After PSM				
	Total (n = 496)	AC (n = 328)	MANEC (n = 168)	Statistic	*P*	Total (n = 380)	AC (n = 251)	MANEC (n = 129)	Statistic	*P*
Age	65.10 ± 8.78	65.30 ± 9.15	64.71 ± 8.00	t=0.705	0.481	64.88 ± 8.75	64.82 ± 9.14	65.02 ± 7.95	t=-0.210	0.834
BMI	22.20 ± 3.35	22.34 ± 3.32	21.93 ± 3.40	t=1.281	0.201	22.31 ± 3.37	22.47 ± 3.39	22.00 ± 3.31	t=1.275	0.203
Sizes				χ²=18.632	<.001				χ²=4.016	0.045
<5	259 (52.22)	194 (59.15)	65 (38.69)			201 (52.89)	142 (56.57)	59 (45.74)		
≥5	237 (47.78)	134 (40.85)	103 (61.31)			179 (47.11)	109 (43.43)	70 (54.26)		
T stage, n (%)				χ²=7.139	0.008				χ²=0.080	0.778
T1+T2	207 (41.73)	123 (37.50)	84 (50.00)			154 (40.53)	103 (41.04)	51 (39.53)		
T3+T4	289 (58.27)	205 (62.50)	84 (50.00)			226 (59.47)	148 (58.96)	78 (60.47)		
Ki-67				χ²=4.168	0.041				χ²=5.037	0.025
<60%	262 (52.82)	184 (56.10)	78 (46.43)			204 (53.4)	147 (57.42)	57 (45.24)		
≥60%	234 (47.18)	144 (43.90)	90 (53.57)			178 (46.6)	109 (42.58)	69 (54.76)		
TNM stage				χ²=0.448	0.503				χ²=0.184	0.668
I+II	217 (43.75)	147 (44.82)	70 (41.67)			168 (44.21)	109 (43.43)	59 (45.74)		
III+IV	279 (56.25)	181 (55.18)	98 (58.33)			212 (55.79)	142 (56.57)	70 (54.26)		
Surgical resection				χ²=0.002	0.966				χ²=0.342	0.559
Incomplete	95 (19.15)	63 (19.21)	32 (19.05)			71 (18.68)	49 (19.52)	22 (17.05)		
Complete	401 (80.85)	265 (80.79)	136 (80.95)			309 (81.32)	202 (80.48)	107 (82.95)		
LMN				χ²=17.384	<.001				χ²=9.228	0.002
No	277 (55.85)	205 (62.50)	72 (42.86)			209 (55)	152 (60.56)	57 (44.19)		
Yes	219 (44.15)	123 (37.50)	96 (57.14)			171 (45)	99 (39.44)	72 (55.81)		
Gender				χ²=9.565	0.002				χ²=1.382	0.240
Female	256 (51.61)	153 (46.65)	103 (61.31)			205 (53.95)	130 (51.79)	75 (58.14)		
Male	240 (48.39)	175 (53.35)	65 (38.69)			175 (46.05)	121 (48.21)	54 (41.86)		
Location				χ²=33.866	<.001				χ²=0.986	0.611
Upper	171 (34.48)	84 (25.61)	87 (51.79)			133 (35)	84 (33.47)	49 (37.98)		
Middle	235 (47.38)	178 (54.27)	57 (33.93)			171 (45)	114 (45.42)	57 (44.19)		
Lower	90 (18.15)	66 (20.12)	24 (14.29)			76 (20)	53 (21.12)	23 (17.83)		
Vascular invasion				χ²=10.405	0.001				χ²=3.355	0.067
No	248 (50)	181 (55.18)	67 (39.88)			181 (47.63)	128 (51.00)	53 (41.09)		
Yes	248 (50)	147 (44.82)	101 (60.12)			199 (52.37)	123 (49.00)	76 (58.91)		
Perineural invasion				χ²=0.311	0.577				χ²=0.449	0.503
No	236 (47.58)	159 (48.48)	77 (45.83)			177 (46.58)	120 (47.81)	57 (44.19)		
Yes	260 (52.42)	169 (51.52)	91 (54.17)			203 (53.42)	131 (52.19)	72 (55.81)		
Distant metastases				χ²=3.550	0.060				χ²=1.721	0.190
No	296 (59.68)	186 (56.71)	110 (65.48)			224 (58.95)	142 (56.57)	82 (63.57)		
Yes	200 (40.32)	142 (43.29)	58 (34.52)			156 (41.05)	109 (43.43)	47 (36.43)		

OR, odds ratio; 95%CI, 95% confidence interval; BMI, body mass index; T stage, tumor stage; Ki-67, Ki-67; Index LMN, lymph node metastasis; AC, adenocarcinoma; NEC, neuroendocrine carcinoma; PSM, propensity score matching.

**Figure 6:**
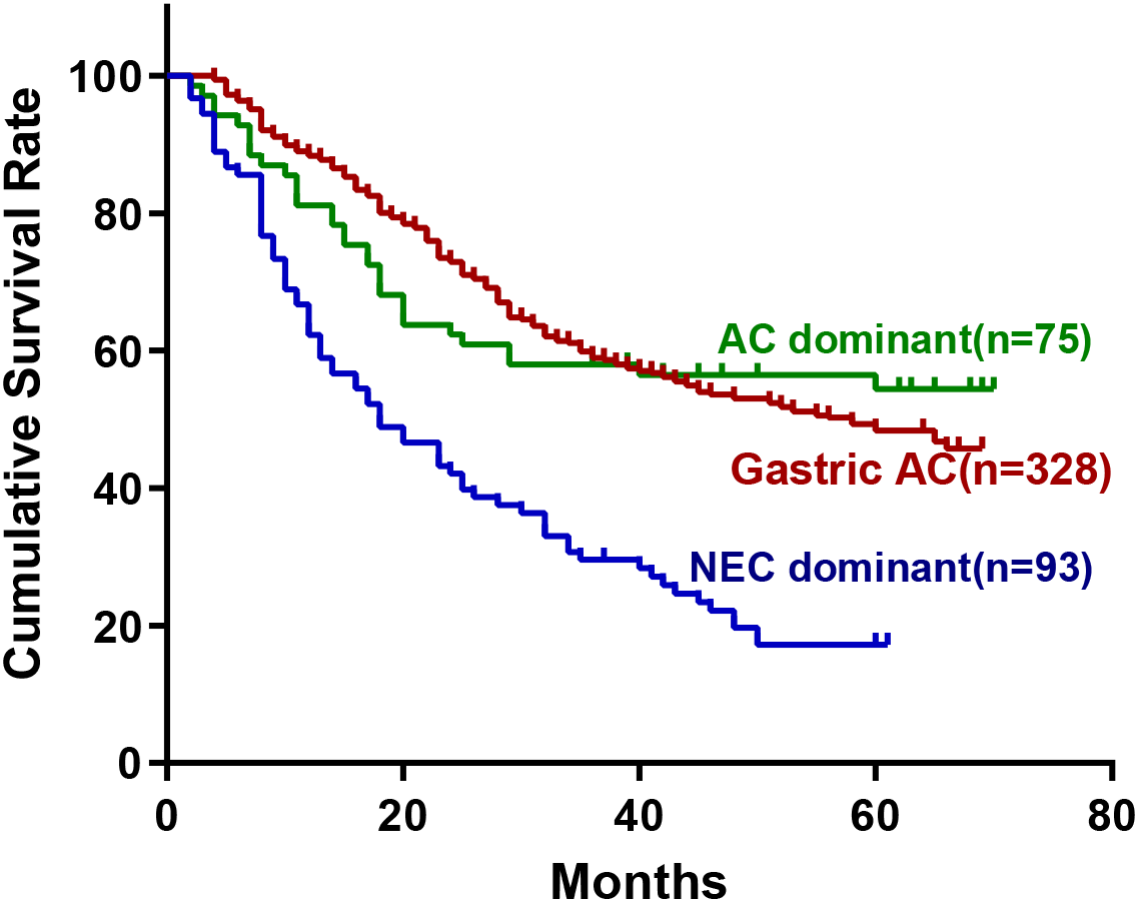
Kaplan–Meier survival curves for gastric cancer patients stratified by histologic subtype. AC dominant, adenocarcinoma-dominant mixed tumors (n = 75); Gastric AC, pure gastric adenocarcinoma (n = 328); NEC dominant, neuroendocrine-carcinoma-dominant mixed tumors (n = 93).

To further elucidate the prognostic heterogeneity within G-MANEC, we performed a stratified survival analysis based on the predominant histological component, comparing NEC-dominant and AC-dominant subtypes with pure adenocarcinoma (AC). No significant difference in survival was observed between AC-dominant G-MANEC and pure AC (HR=1.040; 95% CI: 0.713–1.516; *P*=0.84). In contrast, NEC-dominant G-MANEC was associated with significantly worse survival compared to both AC (HR=0.401; 95% CI: 0.281–0.572; *P* < 0.001) and AC-dominant subtypes (HR=2.423; 95% CI: 1.650–3.559; *P* < 0.001). These findings highlight marked intragroup prognostic divergence and indicate that the unfavorable prognosis of the overall G-MANEC cohort is primarily attributable to the NEC-dominant subtype (**Figure 6**).

## Discussion

Cornier first reported a gastrointestinal carcinoma exhibiting both neuroendocrine and exocrine differentiation in 1924 (21). Since then, this compound tumor has been described with a diverse range of exocrine and neuroendocrine combinations, demonstrating varying degrees of differentiation. In 1987, Lewin proposed classifying these neoplasms into three subtypes-amphicrine tumors, collision tumors, and combined tumors-based on their distinct histopathologic patterns (22, 23). According to the WHO Classification of 2010, these mixed carcinomas are referred to as MANECs, characterized by the presence of both neuroendocrine carcinoma and adenocarcinoma, with each component constituting more than 30% of the tumor (24, 25). Due to the rarity of this subset and the 30% threshold for its mixed nature, much remains to be understood regarding the clinicopathological features and prognosis of MANECs.

In the present study, we identified several clinicopathological characteristics of MANEC that may be significantly associated with poor prognosis. The univariate analysis indicated that the tumor size, differentiation, Ki-67 index, T stage, TNM stage, lymph node metastasis, tumor component were significantly associated with the prognostic of MANEC patients. Multivariate analysis revealed that tumor size, LNM, and tumor component are the independent risk factors for a 3-year survival rate. Our findings further demonstrate that the proportions and histological types of the two components can influence prognosis: patients in the NEC-dominant group experienced significantly worse outcomes compared to those in the AC-dominant group. Additionally, the PDA/NEC type exhibited significantly worse outcomes than the MC and DA type, while no statistical significance was observed between the outcomes of patients with large cell NEC and small cell NEC. Furthermore, this is the first case series to reveal that MANEC differs markedly from CA in terms of clinicopathological features, with MANEC patients showing significantly worse outcomes than those with AC.

Recently, several studies with relatively large cohorts of MANEC patients have been conducted. These studies indicate that MANEC predominantly occurs around the age of 60 and exhibits a clear male predominance. In our study, the mean age was 64.7 years, and the number of male patients was approximately 1.5 times that of female patients. It was consistent with the previous research. In addition, we found that half of the carcinoma located in the upper third of stoma. Recently, most of the studies suggested that the most prevalent primary tumor site was the cardia or the upper part of the stomach (7, 10, 26, 27). Some scholars believe that this may be related to the neuroendocrine carcinoma component in the tumor, but further research is needed (28).

The T-stage of the TNM classification for gastrointestinal cancer is widely regarded as a fundamental indicator of tumor invasion depth (29, 30). However, its appropriateness as a definitive representation of the biological characteristics of the primary tumor in MANEC remains unclear. In 2006, the European Neuroendocrine Tumor Society (ENETS) published a consensus on the TNM staging classification and grading system for NET, which emphasized that T stage should consider not only the depth of invasion but also tumor size (31, 32). Relying solely on tumor invasion to evaluate the biological features of the primary tumor may lead to a distorted understanding. Many studies suggest that the tumor size is a crucial and accurate prognostic factor for detecting and assessing the malignancy of gastric cancer (33, 34). Some research has revealed that tumor size may be more reliable than the depth of invasion in the TNM classification of gastric cancer (35). In our study, we found that tumor size is an independent risk factor for MANEC, while tumor invasion is not. Thus, we propose that both tumor invasion and size should be incorporated into the T categories of the TNM classification for MANEC. This inclusion may enhance the prognostic accuracy of the TNM staging system for MANEC. However, some researchers maintain that the tumor size is an inappropriate independent prognostic indicator due to the lack of global consensus on optimal cutoff intervals for tumor sizes (36). For instance, Guiliani et al. reported that tumor size could be categorized using cutoff points of 2.5cm and 5cm, both showing significant differences in prognosis, while Ahmet Bilici proposed an 8cm cutoff point (37, 38). We choose a cutoff point for the tumor diameter of 5cm, and found that patients with larger tumor size show a significantly worse outcome than that of the patients with smaller size. Therefore, several clinical guidelines recommend considering tumor size as a critical factor in staging gastrointestinal tumors, which aids in more accurately assessing disease severity and treatment options.

We further investigated whether lymph node metastasis is an indicator for predication the prognosis of MANEC. The result showed that MANEC seemed to have more frequent incidences of LNM, which was then indicated as the independent risk factor for the outcomes of MANEC. In addition, we identified the metastasis component of the positive lymph nodes in every patient. In fact, most pathologists report the infiltration of lymph nodes rather than their composition (39). They often do not provide detailed descriptions of the metastatic components and their differentiation levels, which undoubtedly results in the loss of critical diagnostic information (40). We identified three patterns of metastasis based on histological type: solely NEC, solely AC, and a coexistence of AC with NEC. Furthermore, distinct metastatic patterns may be associated with differing prognoses. Patients with LNM with AC demonstrated a significantly more favorable prognosis compared to those with NEC and NEC/AC metastasis. Xie et al. analyzed 597 postoperative lymph nodes in 80 MANEC patients. The results showed that in 89% of the AC group patients, 223 (82.9%) were classified as pure AC, 34 (12.6%) as pure NEC, and 12 (4.5%) as mixed components. In the NEC group, 297 (90.5%) were identified as pure NEC, 26 (7.9%) as pure AC, and only 5 (1.6%) as mixed components (7). This analysis highlights the predominant histological types present in MANEC patients and suggests variations in lymph node involvement between different tumor types. The fact that MANEC represents a different component proportion, histologic subtypes and a wide range of tumor grade makes it difficult to evaluate the biological characteristics and prognosis of the heterogeneous tumor (19). Some research suggested that the biological feature of G-MANEC mainly depends on the proportion of tumor components (more than 50%) (7). And some research revealed that the more aggressive component or the higher degree of malignant one may determine the outcomes (41). Levi Sandri et al. argue that the characteristics of the neuroendocrine component significantly influence the clinical behavior of gastro-enteropancreatic MANEC. They point out that neuroendocrine carcinoma is typically poorly differentiated and more aggressive than adenocarcinoma (42, 43). In our study, many of the tumors were the NEC dominant type, and patients with this type showed a significantly worse outcome than those of patients with the AC dominant type, which suggested that tumor component is another independent risk factor for MANEC. These results were consistent with the previous reports. Moreover, based on histological differentiation, the neuroendocrine carcinoma (NEC) component can be categorized into large cell neuroendocrine carcinoma and small cell neuroendocrine carcinoma. Additionally, the adenocarcinoma (AC) component can be classified into three subgroups: differentiated adenocarcinoma (DA), mucinous carcinoma (MC), and poorly differentiated adenocarcinoma (PDA) (9). Our study further demonstrates that approximately 70% of the NEC cells are classified as large cells. Importantly, there were no significant differences in the 3-year survival rates between the large cell and small cell groups. However, the 3-year survival rate of the AC/NEC group was higher than that of both the MC and PDA groups, while the PDA group exhibited significantly worse outcomes compared to the MC group. This conclusion suggests that the poorly differentiated glandular component may drive cancer progression in MANEC. Recently, an increasing number of pathologists have proposed two main theories regarding the histological origin of MANEC. The prevailing view is that the composite carcinomas of MANEC originate from a common precursor (44). Some histological and immunohistochemical analyses suggest that the NEC component is derived from the preceding AC component, indicating that it represents the neuroendocrine phenotype of dedifferentiated adenocarcinoma (45, 46). Conversely, Bakkelund revealed that signet ring cell carcinomas arise from the gradual dedifferentiation of ECL cells, which are the primary neuroendocrine cells of the stomach (47, 48). Additionally, some researchers propose that the two cellular components originate from multipotent stem cells with bidirectional differentiation (49). Ling Nie argues that most MANECs (DA/NEC) are precursor lesions of NECs, while MANECs (PDA/MC) may develop directly from amphicrine carcinoma, representing a terminal differentiation stage (19). In our study, the finding that NEC has a poorer prognosis than MANEC supports this hypothesis.

Recent findings by Metovic et al. identified four molecular subtypes (A/N/P/Y) of extra-pulmonary NECs based on the expression of lineage-specific transcription factors: ASCL1, NEUROD1, POU2F3, and YAP1. In MiNENs, these markers were detected exclusively in the NEC component, whereas the non-neuroendocrine component was typically negative or YAP1-positive. This supports the hypothesis of distinct clonal origins and divergent molecular pathways between the two components (50, 51). These results underscore the importance of component-specific sampling in MiNENs to enable accurate molecular subtyping. Such classification may offer a more rational basis for treatment stratification, moving beyond traditional morphology-based approaches.

It has been reported that the expression of Ki-67 was significantly up-regulated in many cancer tissues, and closely correlated with increased invasion, proliferation, and poor outcomes (52, 53). In 2004, Ki-67 was defined as prognostic marker or factors according to the WHO classifications of gastroenteropancreatic neuroendocrine neoplasms(GEP-NENs) (54). And then, GEP-NENs were classified into the three-tiered grading mainly based on the Ki-67 index. Boo et al. found that patients with higher Ki-67 index (> 60%) showed higher recurrence rates and worse histological differentiation (55). Sorbye et al. demonstrated that a higher Ki-67 index was associated with higher responsiveness to platinum-based chemotherapy but a poor outcome. In our study, we used the 60% as the cut-off point and found that higher Ki-67 expression had a trend of significant correlation with poor 3-year survival rate, but the multivariate analysis suggested that Ki-67 index is not an independent prognostic factor for MANEC.

This study has several limitations. First, its retrospective design introduces selection bias, as it relies on historical data that may not fully represent the broader patient population. Second, the data were primarily collected from Eastern countries, raising concerns about the generalizability of the findings to other populations. The lack of external validation, particularly from Western cohorts, limits the ability to confirm these results across diverse demographic settings. To enhance the robustness and applicability of these findings, multi-center prospective studies with larger sample sizes are needed.

## Conclusion

In this study, we analyzed 168 cases of gMANEC and demonstrated that the relative proportions of AC and NEC components directly influence clinical outcomes. Independent predictors of three-year survival included tumor size, LNM, and histological subtype (AC/NEC). Furthermore, G-MANEC exhibited significant clinicopathological differences compared to gastric adenocarcinoma, particularly in tumor size, Ki-67 index, LNM. G-MANEC also showed distinct pathological divergence from pure AC, with a markedly poorer prognosis. These findings establish a critical association between histological composition and survival outcomes. By complementing existing prognostic frameworks, our results support the integration of histological stratification into clinical management protocols. Additionally, a systematic evaluation of metastatic involvement should be prioritized to guide therapeutic decision-making and optimize patient-specific treatment strategies.

## Data Availability

The raw data supporting the conclusions of this article will be made available by the authors, without undue reservation.
