# Supplementary material for: Insights into gastric mixed adenoneuroendocrine carcinoma: a novel comparative study of clinicopathological features and survival outcomes
Source: Front Endocrinol (Lausanne). 2025 Sep 30;16:1650314. doi: 10.3389/fendo.2025.1650314 (PMC12518066; doi:10.3389/fendo.2025.1650314)
Supplement: Supplementary file 1 [file Table1.docx]

**Supplemental table 1**

| **Supplemental table 1. Analysis of Basic Characteristics and Differences in 1 year** | | | | | |
| --- | --- | --- | --- | --- | --- |
| **Variables** | **Total (n = 168)** | **Survival Status(1 year)** | | **Statistic** | ***P*** |
|  |  | **Yes (n=122)** | **No (n=46)** |  |  |
| **Age** | 64.71 ± 8.00 | 64.79 ± 8.03 | 64.50 ± 8.00 | t=0.21 | 0.84 |
| **BMI** | 21.93 ± 3.40 | 21.91 ± 3.33 | 21.98 ± 3.63 | t=-0.12 | 0.91 |
| **Sizes** |  |  |  | χ²=1.63 | 0.20 |
| **<5** | 68 (40.48) | 53 (43.44) | 15 (32.61) |  |  |
| **≥5** | 100 (59.52) | 69 (56.56) | 31 (67.39) |  |  |
| **T stage, n (%)** |  |  |  | χ²=0.19 | 0.66 |
| **T1+T2** | 83 (49.40) | 59 (48.36) | 24 (52.17) |  |  |
| **T3+T4** | 85 (50.60) | 63 (51.64) | 22 (47.83) |  |  |
| **Ki67** |  |  |  | χ²=0.67 | 0.41 |
| **<60%** | 78 (46.43) | 59 (48.36) | 19 (41.30) |  |  |
| **≥60%** | 90 (53.57) | 63 (51.64) | 27 (58.70) |  |  |
| **TNM stage** |  |  |  | χ²=0.00 | 0.95 |
| **I+II** | 70 (41.67) | 51 (41.80) | 19 (41.30) |  |  |
| **III+IV** | 98 (58.33) | 71 (58.20) | 27 (58.70) |  |  |
| **Surgical resection** |  |  |  | χ²=0.11 | 0.74 |
| **Incomplete** | 136 (80.95) | 98 (80.33) | 38 (82.61) |  |  |
| **Complete** | 32 (19.05) | 24 (19.67) | 8 (17.39) |  |  |
| **Tumor component** |  |  |  | χ²=11.01 | **<.01** |
| AC | 75 (44.64) | 64 (52.46) | 11 (23.91) |  |  |
| NEC | 93 (55.36) | 58 (47.54) | 35 (76.09) |  |  |
| **LMN** |  |  |  | χ²=7.27 | **<.01** |
| **No** | 72 (42.86) | 60 (49.18) | 12 (26.09) |  |  |
| **Yes** | 96 (57.14) | 62 (50.82) | 34 (73.91) |  |  |
| **Gender** |  |  |  | χ²=1.29 | 0.26 |
| **Female** | 103 (61.31) | 78 (63.93) | 25 (54.35) |  |  |
| **Male** | 65 (38.69) | 44 (36.07) | 21 (45.65) |  |  |
| **Location** |  |  |  | χ²=0.61 | 0.74 |
| **Upper** | 87 (51.79) | 62 (50.82) | 25 (54.35) |  |  |
| **Middle** | 57 (33.93) | 41 (33.61) | 16 (34.78) |  |  |
| **Lower** | 24 (14.29) | 19 (15.57) | 5 (10.87) |  |  |
| **Vascular invasion** |  |  |  | χ²=0.34 | 0.56 |
| **No** | 67 (39.88) | 47 (38.52) | 20 (43.48) |  |  |
| **Yes** | 101 (60.12) | 75 (61.48) | 26 (56.52) |  |  |
| **Perineural invasion** |  |  |  | χ²=0.52 | 0.47 |
| **No** | 77 (45.83) | 58 (47.54) | 19 (41.30) |  |  |
| **Yes** | 91 (54.17) | 64 (52.46) | 27 (58.70) |  |  |
| **Distant metastases** |  |  |  | χ²=0.17 | 0.68 |
| **No** | 110 (65.48) | 81 (66.39) | 29 (63.04) |  |  |
| **Yes** | 58 (34.52) | 41 (33.61) | 17 (36.96) |  |  |
| t: t-test, χ²: Chi-square test; | | | | | |
| SD: standard deviation | | | | | |
